# The Pharmacological Potential of Novel Melittin Variants from the Honeybee and Solitary Bees against Inflammation and Cancer

**DOI:** 10.3390/toxins14120818

**Published:** 2022-11-22

**Authors:** Pelin Erkoc, Björn Marcus von Reumont, Tim Lüddecke, Marina Henke, Thomas Ulshöfer, Andreas Vilcinskas, Robert Fürst, Susanne Schiffmann

**Affiliations:** 1 Institute of Pharmaceutical Biology, Faculty of Biochemistry, Chemistry and Pharmacy, Goethe University Frankfurt, 60438 Frankfurt, Germany; 2 LOEWE Center for Translational Biodiversity Genomics (LOEWE-TBG), Senckenberganlage 25, 60325 Frankfurt, Germany; 3 Applied Bioinformatics Group, Faculty of Biological Sciences, Goethe University Frankfurt, Max-von-Laue-Str. 13, 60438 Frankfurt, Germany; 4 Department of Bioresources, Fraunhofer Institute for Molecular Biology and Applied Ecology (IME-BR), 235394 Giessen, Germany; 5 Fraunhofer Institute for Translational Medicine and Pharmacology (ITMP), 60596 Frankfurt, Germany

**Keywords:** venom, melittin, melittin variants, honeybee, solitary bees, inflammation, anti-tumor effect

## Abstract

The venom of honeybees is composed of numerous peptides and proteins and has been used for decades as an anti-inflammatory and anti-cancer agent in traditional medicine. However, the bioactivity of specific biomolecular components has been evaluated for the predominant constituent, melittin. So far, only a few melittin-like peptides from solitary bee species have been investigated, and the molecular mechanisms of bee venoms as therapeutic agents remain largely unknown. Here, the preclinical pharmacological activities of known and proteo-transcriptomically discovered new melittin variants from the honeybee and more ancestral variants from phylogenetically older solitary bees were explored in the context of cancer and inflammation. We studied the effects of melittin peptides on cytotoxicity, second messenger release, and inflammatory markers using primary human cells, non-cancer, and cancerous cell lines. Melittin and some of its variants showed cytotoxic effects, induced Ca^2+^ signaling and inhibited cAMP production, and prevented LPS-induced NO synthesis but did not affect the IP3 signaling and pro-inflammatory activation of endothelial cells. Compared to the originally-described melittin, some phylogenetically more ancestral variants from solitary bees offer potential therapeutic modalities in modulating the in vitro inflammatory processes, and hindering cancer cell viability/proliferation, including aggressive breast cancers, and are worth further investigation.

## 1. Introduction

Venoms are complex chemical systems and are predominantly composed of proteins and peptides [1]. They evolved convergently in all major lineages of the animal kingdom and are employed for three major objectives: (i) defense, (ii) predation, and (iii) intraspecific competition plus several minor functions [2,3]. Under the influence of millions of years of adaptation, many components (toxins) in such venoms were functionally optimized. They were thereby turned into potent biomolecules, capable of exerting their damaging effects with great efficiency. Several toxins also interfere with pharmacologically relevant targets. Thus, venoms are widely recognized as a prolific source of novel drug leads [4,5].

The mega-diverse hymenoptera are known for their ecologically important role as pollinators [6]. Besides this important function, hymenopterans are also the animal group that harbors the most venomous species. In particular, the aculeates (bees, wasps, and ants) that evolved the characteristic waist in combination with the stinger as a modified ovipositor that is solely used for predation or defense are notoriously recognized to employ venom [7,8]. Since aculeates and especially honeybees (*Apis* spp.) occur in close proximity to humans and their stings can cause severe, sometimes fatal allergic reactions, it comes as no surprise that some aculeates are among the best-studied venomous animals. In particular, the allergenic mechanisms and envenomation effects of honeybee venom components have been well studied for decades [9,10]. The beneficial effects of honeybee venom have been known since antiquity, and the venom is widely used in traditional medicine as an anti-inflammatory, anti-infective, and recently, anti-tumor agents [8,9,10,11].

Honeybee venom is a surprisingly complex mixture that is dominated by short peptides, especially melittin and apamin, but it also contains larger proteins and enzymes, such as phospholipase A_2_, hyaluronidase, and venom allergens. Probably, the most studied bee toxin is melittin (Melt), which is the main component of the honeybee venom that accounts for about 52% of its dry mass. It is synthesized as an inactive precursor (prepromelittin), which consists of 70 amino acids. The precursor can be processed into melittin in a multi-step process [12]. Melittin is widely known for its various pharmacological actions, such as pore-forming, hemolyzing, antibacterial, antifungal, antiviral, and anti-cancer activities [5,11,13,14,15,16]. However, it also displays severe cytotoxicity against other cell types, likely rendering melittin too toxic for applications in humans [17]. Thus, to reduce toxic effects on healthy cells and increase tumor cell selectivity, novel application strategies, including the encapsulation of melittin in nanocarriers, have been developed [5,18].

Likewise, another promising strategy to enhance the therapeutic potential of melittin might be a phylogeny-based multi-omics approach. Variants of melittin genes and peptides in earlier lineages of bees (*sensu lato*) could be less toxic and show novel or different activities compared to melittin from *Apis* spp. and could possibly be identified by this approach. However, the venoms of early bee lineages have received little attention thus far. Only a small subset of species has been studied, and few analyses have been conducted focusing on solitary bee species to identify and test anti-microbial peptides. Recently, the venoms of the solitary bees *Xylocopa violacea* and *Halictus scabiosae* (and complementary *Apis mellifera*) were proteo-transcriptomically analyzed in combination with comparative genomics to unravel the evolution of prevalent bee venom genes [7,19]. Interestingly, most of the small peptides identified in the solitary bees such as halictin (Hal), codesane (Cod), xylopin (Xac), melectin (Mel), and osmin (Osm) were identified as variants of melittin, which is a toxin family unique to bees *sensu lato* [7]. However, the bioactivity of these peptides and thus their pharmacological potential has not yet been elucidated. We focused on melectin from the mourning bee (*Melecta albifrons*), various xylopins from the Japanese and violet carpenter bee (*Xylocopa appendiculata, Xylocopa violacea*), osmin from the mason bee (*Osmia bicornis*), codesane from the silk bee (*Colletes daviesanus*), halictins from sweat bees (*Halictus sextinctus*), macropin (Mac) from the oil bee (*Macropis fulvipes*), and lasioglossins (Las) from the sweat bee (*Lasioglossum laticeps*).

This study aimed to investigate the pharmacological profile of several of these pharmacologically not yet well-characterized variants of melittin peptides in the context of inflammation and cancer and to predict the impact of single amino acids, terminal peptides, sequence alterations, and truncations on their bioactivity. For that purpose, the cytotoxicity of these peptides was studied and their effects on second messenger release and inflammatory markers were addressed.

## 2. Results

### 2.1. Effects of Melittin Peptides on Cell Viability

First, we studied the activity of the melittin peptides on the viability of non-cancer cells. The toxicity of all melittin variants was investigated in monocyte/macrophage-like cells (RAW264.7), human embryonic kidney 293 cells (HEK293T), human microvascular endothelial cells (HMEC), and primary human umbilical vein endothelial cells (HUVECs).

**Figure 1:**
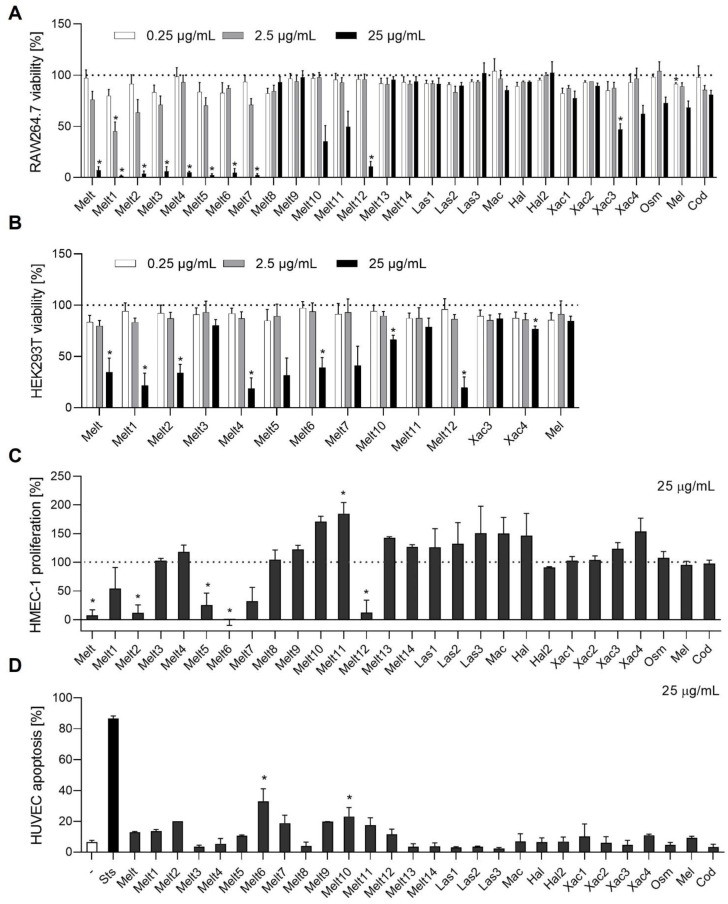
Toxicity characterization of cells treated with melittin peptides. (**A**,**B**) For the viability assay, RAW264.7 (**A**) or HEK293T (**B**) cells were treated with bee venom compounds in the indicated concentrations for 24 h. Cells were incubated with WST-8 and the formed formazan was detected by absorbance measurements. (**C**) For the proliferation assay, HMEC-1 cells were grown in low density and treated after 24 h with the indicated peptide (25 µg/mL) for 72 h. Cells were stained with crystal violet solution. The amount of DNA-bound crystal violet was detected by absorbance measurements. (**D**) The number of sub-diploidic cells was determined by staining with propidium iodide (PI) and quantified to assess apoptosis of HUVECs. Data are expressed as mean ± SEM. (**A)** n = 3, (**B**) n = 4, **(C)** n = 2–3, **(D)** n = 2. * *p* ≤ 0.05 versus control.

The viability of RAW264.7 and HEK293T cells was detected by a formazan-based assay. Interestingly, melittin (Melt) and some of its derivatives (Melt1–7, 12, Xac3) reduced cell viability by more than 90% at a concentration of 25 µg/mL (Figure 1A). Only cytotoxic melittin peptides were tested regarding their effect on viability in HEK293T cells. Melt and its derivatives (Melt1, 2, 4–7, 10, 12, Xac4) showed cytotoxicity at 25 µg/mL in HEK293T cells (Figure 1B). The proliferation of HMECs was evaluated by measuring the number of crystal violet-stained cells (Figure 1C). We found that the proliferation was hindered upon treatment with Melt and its derivatives, Melt2, 5–6, and 12, at a concentration of 25 µg/mL for 72 h. However, the other melittin peptides did not decrease the proliferation under the same treatment conditions. Instead, the proliferation was augmented upon Xac4 treatment. The apoptotic (sub-diploid) populations of HUVECs were analyzed by flow cytometry using propidium iodide (PI) staining (Figure 1D). Among the derivatives, Melt6 and 10 caused an increase in the apoptosis level of HUVECs up to 30–40%.

### 2.2. Melittin Peptides Interact with Ca^2+^ and cAMP Signaling in HEK293T Cells

**Figure 2:**
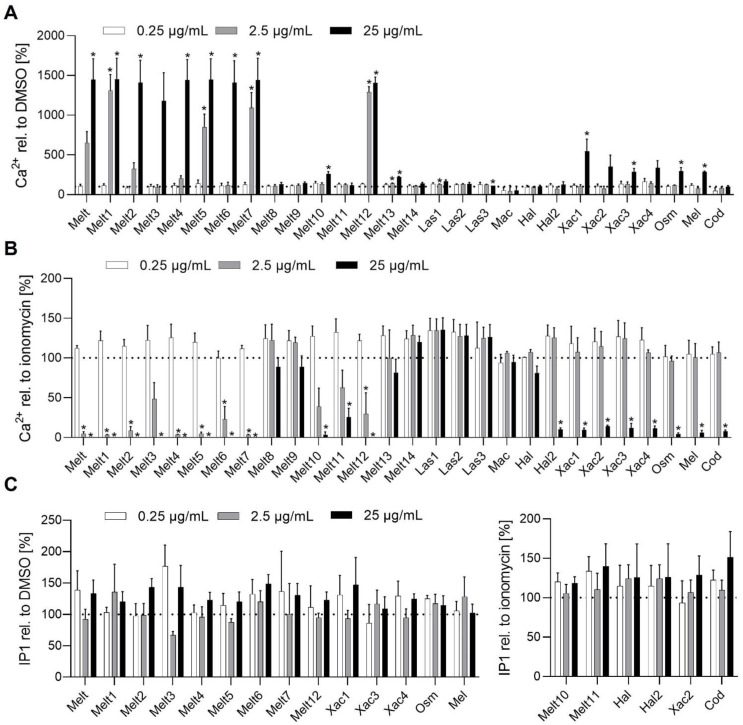
Melittin derivates influence cellular Ca^2+^ and IP1 levels. (**A**) For the Ca^2+^ induction assay, Fluo-8 pretreated HEK293-T cells were incubated with melittin derivates in the indicated concentrations and the fluorescence was detected. (**B**) For the Ca^2+^ inhibition assay, Fluo-8 pretreated HEK293-T cells were incubated with peptides in the indicated concentrations for 30 min, stimulated with 5 µM ionomycin, and the fluorescence was detected. (**C**) IP3 has a short lifetime and is rapidly degraded to IP1; therefore, IP1 was determined. For the IP1/IP3 induction assay (left panel), HEK293T cells were incubated with melittin peptides in the indicated concentration for 2 h, a detection reagent was added, and the fluorescence resonance energy transfer signal was detected. For the IP1/IP3 inhibition assay (right panel), HEK293T cells were incubated with melittin peptides in the indicated concentration for 30 min and stimulated with 20 µM ionomycin for 1.5 h. After the addition of the detection reagent, the fluorescence resonance energy transfer signal was detected. Data are expressed as mean ± SEM. (**A**, **B**, **C** (**left**)) n = 4, (**C** (**right**)) n = 3. * *p* ≤ 0.05 versus control.

To assess the interaction of melittin peptides with cellular signaling processes, we investigated their effect on the intracellular concentration of the second messenger Ca^2+^, inositol 1,4,5-trisphosphate (IP3), and cyclic adenosine monophosphate (cAMP) that play a role in G protein-coupled receptor signaling [20]. Melt and its derivatives (Melt1, 2, 4–7, 10, 12, 13, Xac3, Osm, Mel) induced a significant release of Ca^2+^ partly already at a concentration of 2.5 µg/mL (Figure 2A). For the Ca^2+^ inhibition assay, melittin peptide-treated HEK293T cells were stimulated with 5 µm ionomycin. Samples already treated by melittin peptides could not elicit further stimulation by ionomycin. Only, Melt11, Hal2, and Cod inhibited the ionomycin-induced Ca^2+^ release without inducing Ca^2+^ release (Figure 2B).

Since Ca^2+^ release can potentially be mediated by IP3 [21], we investigated if the melittin peptides that release Ca^2+^ also induce IP3 synthesis. However, the tested melittin peptides did not augment IP3 concentrations (Figure 2C). Melittin peptides, which potentially inhibit Ca^2+^ release, were further investigated for potential inhibition of IP3 synthesis. However, the tested melittin peptides did not reduce IP3 synthesis (Figure 2C). These data indicate that some of the melittin peptides modify Ca^2+^ release, which is not linked to IP3 signaling.

**Figure 3:**
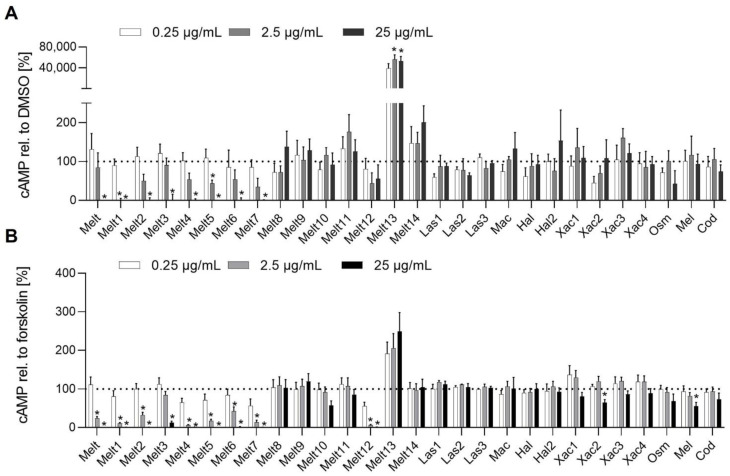
Melittin derivates alter the cellular concentration of cyclic adenosine monophosphate (cAMP). (**A**) For the cAMP induction assay, HEK293T cells transfected with pGloSensor-22F cAMP plasmid were treated with melittin derivates (0.25, 2.5, 25 µg/mL) in presence of the pGloSensor cAMP reagent, and luminescence was detected. (**B**) For the cAMP inhibition assay, HEK293T cells transfected with pGloSensor-22F cAMP plasmid were treated with melittin derivates (0.25, 2.5, 25 µg/mL) in presence of the pGloSensor cAMP reagent, stimulated with 5 µM forskolin, and luminescence was detected. Data are expressed as mean ± SEM. n = 4, * *p* ≤ 0.05 versus control.

Next, we addressed the question of whether melittin peptides interact with cAMP signaling. Most important, Melt13 increased cAMP levels already at 0.25 µg/mL (Figure 3A). Melt and most of its derivatives (Melt1–7, 12) almost completely prevented cAMP synthesis at a concentration of 2.5 and 25 µg/mL in unstimulated and forskolin-stimulated HEK293T cells (Figure 3A,B).

### 2.3. Melittin Peptides Inhibit NO Release in RAW264.7 Cells

**Figure 4:**
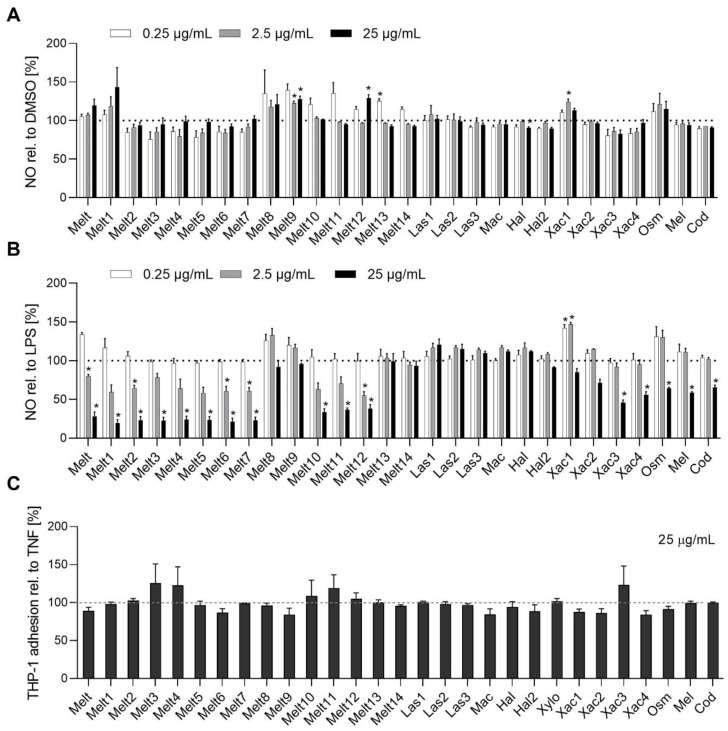
Influence of melittin derivates on inflammation-associated cell functions. (**A**,**B**) For the NO induction assay (**A**), RAW264.7 cells were treated with melittin peptides in the indicated concentrations for 24 h. For the NO inhibition assay (**B**), RAW264.7 cells were pretreated with melittin peptides in the indicated concentrations for 30 min and then stimulated with 100 ng/mL LPS for 24 h. NO was detected in the supernatant using the Griess method. (**C**) Melittin peptides do not interfere with the adhesion of leukocytes on endothelial cells. THP-1 cell adhesion under static conditions. HUVECs were grown to confluence, preincubated with bee venoms for 30 min, and activated with TNF (10 ng/mL) for 24 h. For the leukocyte adhesion assay, untreated THP-1 cells were stained with CellTracker Green and were allowed to adhere to the treated HUVECs for 5 min. The adhesion of leukocytes onto Ecs was quantified by fluorescence measurements for all cell adhesion experiments. Data are expressed as means ± SEM, (**A**,**B**) n = 3, (**C**) n = 2. * *p* ≤ 0.05 vs. TNF (positive) control.

Next, we investigated whether melittin peptides affect inflammatory processes. For that purpose, we tested if they interact with lipopolysaccharide (LPS)-induced inflammatory processes in murine macrophages (RAW264.7), such as the synthesis of nitric oxide (NO). The melittin peptides did not induce NO release (Figure 4A). However, melittin and some of its derivatives (Melt1–7, 10–12, Xac3, Xac4, Osm, Mel, Cod) prevented LPS-induced NO synthesis in a concentration-dependent manner (Figure 4B). The IP3, cAMP, and Ca^2+^ results together with the NO data indicated that Melt8, Melt9, and Melt14 were inactive in comparison to the other Melt derivatives.

### 2.4. Melittin Peptides Did Not Reduce the Adhesion of Leukocytes onto Ecs

The extravasation of leukocytes from the blood into the inflamed tissue is a critical stage of the inflammatory response, and the adhesion of leukocytes onto the vascular endothelium is a key requirement for this extravasation [22]. Hence, we tested melittin peptides for their ability to alter the adhesion of human monocytic (THP-1) cells onto a TNF-activated EC monolayer. As shown in Figure 4C, none of the derivatives diminished the interaction between ECs and leukocytes, and consequently the pro-inflammatory activation of ECs.

### 2.5. Anti-Proliferative and Apoptotic Effect of Melittin Peptides on MDA-MB-231 Cells, a Triple-Negative Breast Cancer Cell Line

**Figure 5:**
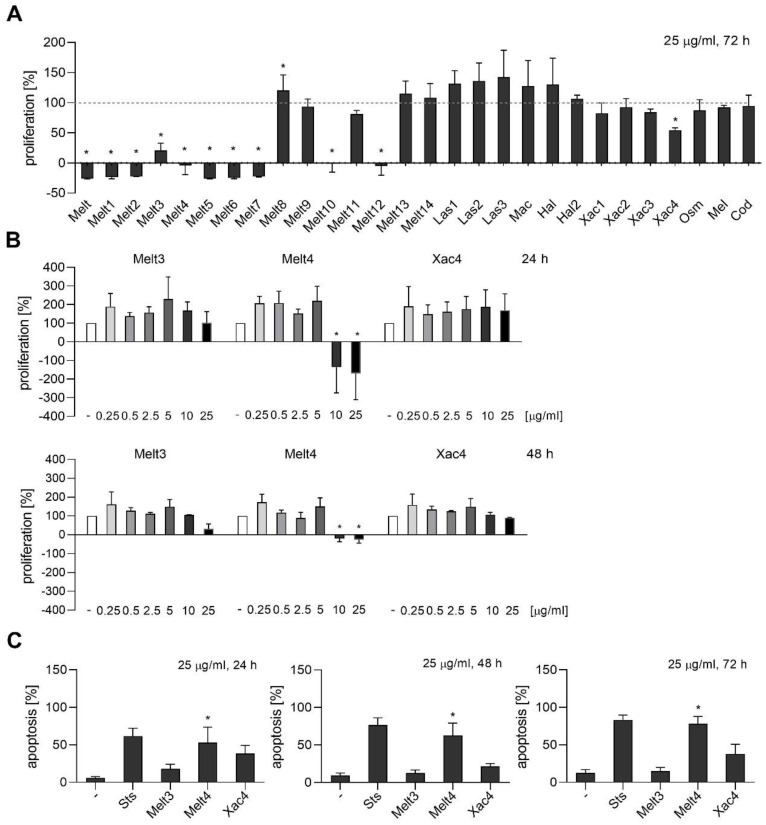
Anti-proliferative and apoptotic effect of melittin derivates on the triple-negative breast cancer cell line MDA-MB-231. (**A**) For the proliferation assay, cells were grown in low density and were treated after 24 h with melittin peptides at a concentration of 25 µg/mL for 72 h. Cells were stained with crystal violet solution. The amount of DNA-bound crystal violet was detected by absorbance measurements. (**B**) Melt3, 4, and Xac4, which are non-toxic to healthy cells, were further used to test effects on proliferation at 24 h (left) and 48 h (right) at different concentrations. (**C**) The effects on apoptosis were detected by flow cytometry after 24 h (left), 48 h (middle), and 72 h (right) of treatment. The number of apoptotic cells was determined by staining with propidium iodide (PI). Data are expressed as mean ± SEM. n = 2, * *p* ≤ 0.05 versus control. Sts, staurosporin 1 µM.

For the proliferation assay, MDA-MB-231 cells were grown in low density and stained with crystal violet solution after 72 h of treatment. As shown in Figure 5A, Melt and Melt1–7, 10, 12, and Xac4 exhibited anti-proliferative activity.

**Figure 6:**
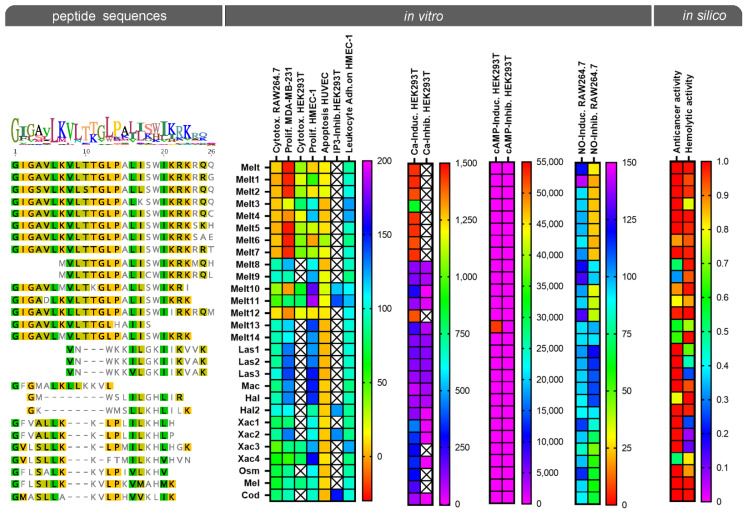
Effects of melittin and its variants on the cytotoxicity (RAW264.7, HEK293T), proliferation (MDA-MB231, HMEC-1), apoptosis (HUVEC), adhesion, Ca^2+^-/cAMP-/IP3-, and NO release visualized in separate heatmaps. In silico predictions for possible hemolytic and general anti-cancer activity are shown as well as applying neuronal network algorithms (ENNAACT and HAPPENN) [23,24]. On the left the sequence similarities of used melittin variants is shown based on aligned mature peptides (green = 100%, olive = 80%, orange = 60–80 and no color = less than 60% similarity). See material and methods for further details.

Melt3, 4, and Xac4, which did not hinder the viability of non-cancer cells (Figure 6), were further used to test effects on proliferation at 24 h (top) and 48 h (bottom) in a concentration range, and apoptosis upon treatment of 24 h (left), 48 h (middle), and 72 h (right) at a concentration of 25 µg/mL. Among them, Melt4 significantly decreased the proliferation of breast cancer cells at concentrations of 10 and 25 µg/mL (Figure 5B). Furthermore, Melt4 was able to induce apoptosis at all tested time points, i.e., after 24 h, 48 h, and 72 h of treatment (Figure 5C). Melt3 and Xac4 also showed a trend of decreased proliferation and increased apoptosis.

## 3. Discussion

Although melittin has long been recognized as an anti-microbial, anti-inflammatory, and anti-cancer agent, its fast degradation in the blood and nonspecific cellular lytic activities are the best-known limiting factors preventing its therapeutic use [17]. Therefore, the identification of biologically active derivatives of melittins with minimal toxicity to healthy cells is important for developing new pharmacological treatment options. In this study, we applied a broad in vitro screening approach of melittin variants from the honeybee and earlier solitary bee lineages to assess whether these are less toxic to healthy human cells and more effective in the context of inflammation and cancer compared to the originally described melittin (Melt).

Our results revealed that Melt and some of its variants, such as Xac4 (Carpenter bees), Osm (Mason bee), and Mel (Honeybee), induce Ca^2+^ release, while variants from earlier solitary bee lineages such as carpenter bees (Xac1, Xac2), plasterer bees (Cod), and sweat bees (Hal) inhibit Ca^2+^ levels. Moreover, most of them reduced cAMP levels, except for Melt13, which increased cAMP levels. These two systems are rarely independent but are often antagonistic, sometimes synergistic, or occasionally redundant [25]. Ca^2+^ is never elevated without a possible consequence—either positive or negative—for cAMP levels emanating from any of the adenylyl cyclases (AC)—the enzymes that catalyze the conversion of ATP to cAMP [26]. Calcium can directly inhibit AC5 and AC6 or indirectly AC1, AC3, and AC9 via the Ca-binding proteins calmodulin-activated kinase (CaMK) and calcineurin [24]. On the other hand, it was reported that Melt is a competitive inhibitor of the calmodulin function [27]. Targets of the Ca^2+^/calmodulin complex are CaMK, ACs, the phosphatase calcineurin, and the inducible NOS [28,29]. Calmodulin can stimulate AC, and therefore, Melt possibly inhibits ACs via calmodulin and the release of cAMP. Interestingly, all these ACs were expressed in a similar amount in HEK293 cells [30]. Though, additional studies are needed to elucidate if one or more of the ACs were involved in the reduced cAMP level and whether the reduced cAMP levels are a consequence of increased Ca^2+^ levels or the inhibition of calmodulin.

In addition, Xac3 from carpenter bees induced significant cytotoxicity and inhibit NO synthesis in RAW264.7 macrophage, a tumor-originating cell line. LPS induces iNOS expression and the release of NO in RAW264.7 macrophages [31]. Melittin was able to prevent the LPS-induced NO release in these cells. iNOS is catalytically active in the presence of CaM but is not regulated by intracellular Ca^2+^ concentrations [28,32]. Thus, it is possible that the inhibition of calmodulin by melittin leads to an inactive iNOS and thereby reduced NO levels. Previously, Tang et al. reported that Melt prevents NF-κB activation by inhibiting both I-κB degradation and NF-κB migration into the nucleus in IL-1β-activated rat knee joint cells. They also demonstrated that the reduced NF-κB activation leads to a reduced iNOS mRNA and protein expression [33]. Melittin also reduced LPS-activated BV2 microglia iNOS expression and NO synthesis by blocking NF-κB translocation [34]. In correspondence with the literature data, our results indicate that melittin reduces the activity of iNOS and/or the expression of iNOS and thereby NO synthesis.

Melt is a protein consisting of 26 amino acids and has the following sequence: GIGAVLKVLTTGLPALISWIKRKRQQ (Figure 6). Compared to the well-known Melt, the other naturally occurring variants have amino acid substitutions in terminal sides and/or inner parts. Among the analyzed peptides, Melt8 and Melt9 were found to exert no cytotoxicity and no strong interaction with the studied cellular functions. Melt8 and Melt9 have a similar peptide sequence, but both lack the “GIGAVL” amino acids at their N-terminal. Melt13 and Melt14 also show no cytotoxic effects and lack the C-terminus “WIKRKRQQ” or “RQQ”, respectively, in comparison to the original Melt. These data suggest that the N- and/or the C-terminal side of Melt is responsible for the cytotoxic effects. Interestingly, Raghuraman found that the C-terminal region of melittin is hydrophilic and responsible for the lytic action, while the N-terminal region of its sequence is predominantly hydrophobic with no lytic activity [35]. Melt10, Melt11, and Melt 14 lack the C-terminal amino acids “RQQ”, but Melt10 and Melt11 reduce viability (by 40%), however to a lesser extent in comparison to melittin (by 90%). These data indicate that the point mutation K7M of Melt10 (K7M, T11K, I17L, K23I) and Melt11 (K7M) contribute to cytotoxicity or that the mutation of Melt14 (V5D) protects from cytotoxicity. The fact that the variants Melt8 and 9 are not cytotoxic, but also carry this mutation K7M, speaks against a role for K7M in cytotoxicity. Otoda et al. found that K23 and R24 are important for the binding of melittin to the polar region of the membrane for lysis [36]. However, Melt10 with the mutation K23I induces cytotoxicity, and the exchange of R24 to R24S (Melt5, 6) is linked to the cytotoxic effect. The mutant R24M (Melt8) showed no cytotoxic effect, but it also lacks the N-terminus “GIGAVL” and has two further mutations, L6M and Q26H. Thus, the lost cytotoxic effect of Melt8 cannot be assigned to a specific mutation, and we could not demonstrate that K23 and R24 are important for the cytotoxic effects of melittin.

Furthermore, melittin displayed significant effects on multiple types of tumor cells by causing more damage to the tumor cell membranes compared to healthy cells [13,17]. For example, in a recent study, melittin derivates and melittin were found to induce apoptosis of the aggressive triple-negative and HER2-enriched breast cancer subtypes by suppressing the activation of EGFR and HER2 receptors by blocking the phosphorylation in the plasma membrane of breast carcinoma cells [16]. It was also reported that a positively charged C-terminal peptide end is responsible for the plasma membrane interaction and anti-cancer activity, as well as that conjugation of an RGD motif further improves the targeting of melittin to malignant cells.

Among the melittin derivative peptides tested in this study, we found several peptides exhibiting better bioactivity than melittin on the triple-negative breast cancer cell (TNBC) line, MDA-MB-231, without harming human umbilical vein endothelial cells (HUVEC) and human microvascular endothelial cells (HMEC-1). This perspective is very important because less toxic melittin variants could be applied more directly and effectively to treat patients with, for example, breast cancer. Recent studies tested to bypass the high toxicity of the original melittin by applying more complex solutions in which melittin is transported to tumor tissues bound to niosome nanoparticles [5,18]. Particularly, Melt4, which has a single amino acid change of glutamine (Gln) to cysteine (Cys) at the C-terminus was successful to induce cancer cell death. The sulfhydryl group at the side chain of the Cys amino acid makes it reactive for a covalent coupling with amino groups, thus, may have conducted the binding of the peptide to the cell membrane surface and the disruption of the cell [37]. However, the potential mechanism of efficiency increase by the addition of a Cys residue to melittin needs to be further investigated. Similarly, Melt3, which carries an I17K substitution also exhibited milder effects on healthy cells but significantly hindered MDA-MB-231 cell proliferation at 72 h. Since K is more polar and hydrophilic than I, this might reduce the passage through the cell membrane. Furthermore, Xac4 from carpenter bees is also promising for exhibiting anti-proliferative effects on breast cancer cells without harming healthy cells and altering the inflammatory processes by reducing the inflammation-associated interaction of endothelial cells with leukocytes and NO synthesis. Though, it only carries small similarities with Melt (“G” at N-terminus and “LK” at 6–7 positions).

Finally, to provide in silico predictions for possible hemolytic and general anti-cancer activity of the peptides, we used artificial neural network models (ENNAACT and HAPPENN) [23,24]. As shown in Figure 6, like the in vitro results, the artificial neural network model ENNAACT also found that the peptides Melt8, 9 and Melt13, 14 were less active against cancer compared to the other Melt variants of honeybee. However, the other predictions diverged from our in vitro findings, which might have resulted from the biologically diverse nature of cancer types. Particularly, the cancer cell line used for the experiments, MDA-MB-231, is a most malignant subtype of breast cancer.

Overall, melittin variants interact with cellular functions in the context of inflammation and cancer but to varying degrees. At this stage, we can only speculate about the biological purpose and activity of these bee unique peptides. It could be that with the evolution of sociality, the activity of melittin known from the honeybee was tailored towards a strong effect to non-insect predators that attack the hive for food (honey and brood) while solitary bees must defend rather against other insect predators. To test plausible evolutionary hypotheses, the different biological targets and bioactivities should be further studied. Nevertheless, the lesser toxic effect of solitary bee melittin variants on several cell lines, in particular, is extremely appealing from an applied perspective for further translational studies.

## 4. Conclusions

In summary, the melittin variants with sequence alterations, and truncations screened in the present study exhibited different activity on different cells, and several of them have significant pharmacological activity in the context of inflammation and/or cancer. In particular, the variants from solitary bees give new perspectives varying in their toxicity accompanied by higher application potential. Our results indicate that this peptide family includes promising members for further testing.

## 5. Materials and Methods

### 5.1. Materials

**Figure 7:**
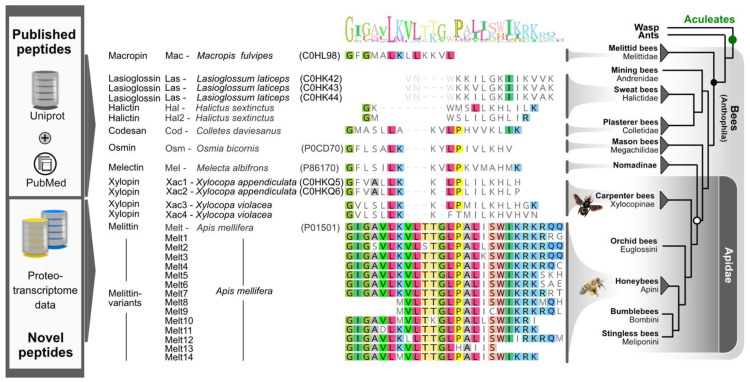
The strategy of phylogeny-based data mining for melittin variants from published and novel data. The sequences were aligned using mafft-L-INSI with standard settings [5,38]. The bee phylogeny is adapted according to Peters et al. [6] and Bossert et al. [39]. Some bee groups were for feasibility pruned and omitted. Please note that Lasioglossins were modified, and the first three amino acids were trimmed (in light grey) to test shorter sequences.

The melittin variants were mined from sequences published in UniProt and accessible publications. Additionally, peptides were selected from novel proteo-transcriptome data generated from the honeybee (Apis mellifera) and the violet carpenter bee (Xylocopa violacea). All mature sequences of the peptides were identified by comparative alignments taking functionally known peptides as anchor sequence (Figure 6) and finally purchased from GenScript (Piscataway, NJ, USA), with a confirmed purity of >75%. Identifiers used for the experiments are shown in Figure 6 and Figure 7.

For more details on available melittin peptides and the processing and availability of the novel proteo-transcriptome data, please see BioProject (PRJNA733472), von Reumont, et al. [5], and Koludarov et al. [7].

### 5.2. Cells and Reagents

HEK293T and RAW246.7 were purchased from DSMZ GmbH (Braunschweig, Germany) or ATCC (Virginia, USA), respectively. The human microvascular endothelial cell line CDC/EU.HMEC-1 (HMEC) was obtained from the Centers for Disease Control and Prevention (CDC, Atlanta, GA, USA). The triple-negative breast cancer cell line, MDA-MB-231 (MDA cells; ACC-732) was purchased from the Leibniz Institute for German Collection of Microorganisms and Cell Cultures (DSMZ, Braunschweig, Germany). Primary human umbilical vein endothelial cells (HUVECs) were isolated from human umbilical cords according to Jaffe et al. [40]. A waiver has been granted for the use of anonymized human material issued by the head of the Research Ethics Committee/Institutional Review Board (REC/IRB) on 15 September 2021 under the reference number W1/21Fü. HEK293T was cultured in Dulbecco’s Modified Eagle Medium (DMEM) and RAW264.7 cells in RPMI medium. These media contained 10% fetal calf serum (FCS), 1% penicillin/streptomycin. HMECs and primary HUVECs were cultured in collagen G (Biochrom AG, Berlin, Germany)-coated 75 cm^2^ flasks in supplemented EC growth medium (ECGM) (PELOBiotech, Martinsried, Germany) supplemented with 10% FCS (Biochrom AG), 100 U/mL penicillin, 100 µg/mL streptomycin, and 2.5 µg/mL amphotericin B (PAN-Biotech, Aidenbach, Germany), and a supplement mixture (PELOBiotech). MDA-MB231 cells were cultured in DMEM (PAN-Biotech) containing 10% FCS, 100 U/mL penicillin, and 100 μg/mL streptomycin. All cells were cultured at 37 °C in a 5% CO_2_ atmosphere. Melittin peptides were dissolved in DMSO, and DMSO concentrations in cells did not exceed 0.1% (*v*/*v*).

### 5.3. Cell Viability Assays

For assessing the cytotoxicity of all peptides, various cell types and assays were utilized. To determine the cell viability of HEK293T and RAW246.7 cells, the Orangu^TM^ assay (Cell Guidance Systems Ltd., Cambridge, UK) was used, as previously described [41]. 2 × 10^5^ HEK293T or 2 × 10^5^ RAW246.7 were seeded in 96-well plates. Different concentrations (0, 0.25, 2.5, 25 µg/mL) of melittin peptides or vehicle (DMSO) were added. After 24 h of incubation at 37 °C and 5% CO_2_, 10 µL of Orangu^TM^ cell counting solution was added and incubated for 60 min. After incubation, absorbance was measured at a wavelength of 450 nm with a reference at 650 nm at an EnSpire 2300 Multimode Plate Reader (Perkin Elmer, Lübeck, Germany). To calculate cell viability, the absorbance of vehicle-treated cells was set to 100%, and the melittin derivates-treated samples were correlated to them.

HMECs (2000 cells/well) were seeded into collagen-coated 96-well plates and grown for 24 h. Then, they were treated with melittin peptides. Treated cells were cultured for 72 h, whereas untreated control cells, directly after 24 h, were fixed with a methanol-ethanol (2:1) solution and washed with PBS before they were stained using a crystal violet solution (20% methanol). Similarly, at the end of incubation time, cells treated with melittin peptides were fixed, stained, and unbound crystal violet was removed by washing with distilled water. Finally, cells were left to air dry, and DNA-bound crystal violet was dissolved using an acetic acid solution (20%, Sigma-Aldrich, Steinheim, Germany). Absorbance was measured at 590 nm using a plate reader (Infinite F200Pro, Tecan, Männedorf, Switzerland). The proliferation percentage was calculated by normalizing to the untreated control (24 h) and compared to the DMSO control (0.03%) of 72 h incubation. Similarly, MDA-MB-231 cells (6000 cells/well) were seeded into 96-well plates, and their proliferation ability under treatment conditions was tested as described above.

Apoptosis of primary HUVECs was detected according to the method of Nicoletti et al. [42]. Cells were treated as indicated and were incubated overnight in the dark in a PBS solution containing propidium iodide (PI) (50 µg/mL; MilliporeSigma, Darmstadt, Germany), sodium citrate (0.1%; Carl Roth, Karlsruhe, Germany), and Triton X-100 (0.1%; MilliporeSigma, Darmstadt, Germany) at 4 °C. The percentage of cells with sub-diploidic DNA content was measured using a FACSVerse flow cytometer (BD Biosciences, Heidelberg, Germany).

### 5.4. Analysis of Intracellular Ca^2+^ Levels

For analysis, 5 × 10^4^ HEK293 cells or 2 × 10^4^ MDA-MB-231 cells were seeded in a 96-well poly-D-lysine plate, incubated at 37 °C for 24 h, and treated with 4 µM Fluo-8-AM in HBSS for 1 h at 37 °C. After 1 h, the Fluo-8/HBSS was replaced by 100 µL HBSS. Five images per second were taken using an ImageXpress Micro Confocal High Content Imaging System (Molecular Device, San Jose, CA, USA). Melittin peptides (0.25, 2.5, and 25 µg/mL), DMSO (negative control), or 5 µM ionomycin (Sigma Aldrich; Taufkirchen, Germany) (positive control) were added to the cells. For the next 20 s, an image was obtained every second. For the inhibition assay, the peptide-treated samples (30 min) were treated with 5 µM ionomycin and for the next 20 s, an image was obtained every second. The data were analyzed with the MetaXpress Software Version 6 (Molecular Devices, San Jose, CA, USA). A threshold of fluorescence intensity was defined using cells before treatment, and all cells with a fluorescence signal above the threshold level were counted. For the induction assay, the number of cells above the threshold in the toxin-treated samples were related to the cells in the DMSO-treated sample. For the inhibition assay, the number of cells above the threshold in the melittin-treated samples were related to the cells in the ionomycin-treated sample.

### 5.5. Analysis of cAMP Levels

HEK293T cells were transfected with pGloSensor-22F cAMP plasmid (E2301, Promega, Walldorf, Germany) using turbofect reagent (Thermofisher Scientific, Frankfurt am Main, Germany). 7 × 10^4^ cAMP transfected HEK293T cells were seeded on a white poly-D-lysine 96 well plate and incubated for 24 h. The supernatant was replaced by DMEM without phenol red supplemented with pGlo sensor cAMP reagent (E1290, Promega, Walldorf, Germany). Induction and inhibition assays were performed in two steps with the same plate. For the induction assay (step one), the luminescence was detected (background, 3 measurements every 5 min) and then the melittin peptides (0.25, 2.5, 25 µg/mL) or 5 µM forskolin were added. The luminescence was detected (3 measurements every 5 min) using the plate reader (Spark, Tecan, Männedorf, Switzerland). For the inhibition assay (step tow), the melittin peptide-treated cells were then incubated with 5 µM forskolin and the luminescence was detected (3 measurements every 5 min). For the induction assay, the luminescence values of the melittin peptide-treated samples were related to the DMSO-treated sample. For the inhibition assay, the luminescence values of the melittin peptide-treated samples were related to the forskolin-treated sample.

### 5.6. IP3 Assay

Since the IP3 lifetime within the cell is very short (less than 30 s) before it is transformed into IP2 and IP1, IP1 instead of IP3 levels were detected [43]. The IP1-one Gq kit von Cisbio (Berlin, Germany) was used and performed as described by the supplier. Lithium chloride (LiCl) in the cell stimulation buffer prevents the degradation of IP1 into myo-inositol. Briefly, 2 × 10^4^ HEK293T cells were seeded in a white 384-well plate. The cells were treated with melittin peptides (0.25, 2.5, 25 µg/mL) or 20 µM ionomycin for 2 h at 37 °C and 5% CO_2_. The detection reagent (IP1 Tb cryptate antibody (donor) and d2 reagent (acceptor)) was added for 1 h at room temperature. The fluorescence resonance energy transfer (FRET) signal was detected using the plate reader (Spark, Tecan, Männedorf, Switzerland). For the inhibition assay, HEK293T cells, pretreated for 30 min with peptides (0.25, 2.5, 25 µg/mL), were incubated additionally with 20 µM ionomycin for 1.5 h. After the addition of the detection reagent and incubation of 1 h at room temperature, the FRET signal was detected. Using a standard curve, the concentration of the produced IP1 was calculated. For the induction assay, the IP1 level of the melittin peptide-treated samples was related to the DMSO-treated sample. For the inhibition assay, the IP1 level of all venom peptide samples was related to the ionomycin-treated sample.

### 5.7. Measurements of Nitrogen Oxide (NO) Levels

2 × 10^4^ RAW264.7 macrophages/well were plated in a 96-well plate and cultured for 24 h at 37 °C. To investigate if the melittin peptides induce nitrogen oxide (NO) synthesis, the melittin peptides, control (DMSO), and positive control (100 ng/mL lipopolysaccharide (LPS)) were added. To evaluate the effect of melittin peptides on inhibiting NO synthesis, the cells were pre-incubated with peptides or control (DMSO) for 30 min before 100 ng/mL LPS were added. After 24 h supernatants were collected and stored at −80 °C.

NO was determined with the Griess method. Briefly, for the standard curve, different concentrations of sodium nitrite (0–50 µM) were prepared in the medium. A total of 80 µL cell supernatant or standard sample were added to a 96-well microplate and subsequently, 20 µL sulfanilamide solution (120 mg sulfanilamide in 30 mL 1 N hydrochloric acid) and 20 µL naphthylenediamine solution (180 mg N-(1-naphthyl)ethylenediamine dihydrochloride in 30 mL water) were added. After 15 min of incubation, the absorbance (540 nm) was measured with the EnSpire Plate Reader (PerkinElmer, Waltham, MA, USA). For the induction assay, the NO levels of the melittin peptides-treated samples were related to the DMSO-treated sample. For the inhibition assay, the NO levels of the melittin peptides-treated cells were correlated with the LPS-treated samples.

### 5.8. Adhesion of Leukocytes to Endothelial Cells

For the investigation of leukocyte adhesion under static conditions, HMEC-1 cells were seeded into 96-well plates and cultured until they became confluent. Later, they were preincubated with melittin peptides for 30 min and then activated with 10 ng/mL TNF for 24 h. For the leukocyte adhesion assay, untreated THP-1 cells were stained with CellTracker Green (Thermo Fisher Scientific, Frankfurt am Main, Germany) and they (3 × 10^4^ cells/well) were allowed to adhere to the treated HMECs for 5 min. Non-adherent THP-1 cells were removed through washing steps. Then, the fluorescence signal from adherent THP-1 cells was measured by a Tecan Infinite F200 Pro microplate reader (Tecan, Männedorf, Switzerland) (excitation: 485 nm, emission: 535 nm).

### 5.9. Statistics

For all statistical calculations, the creation of graphs and heat map (Figure 6), GraphPad Prism 8 or GraphPad Prism 9.3.1 (GraphPad, San Diego, CA, USA) was used. Results are presented as mean ± standard error of the mean (SEM). The numbers of independently performed experiments (n) are stated in the corresponding figure captions. The data were analyzed with one-way or two-way ANOVA with Dunnett’s multiple comparisons and Tukey’s post hoc tests. *P* < 0.05 was considered the threshold for significance.

### 5.10. In Silico Predictions

In silico predictions to test for potential anti-cancer and hemolytic activities were performed using two recent tools that apply neuronal network algorithms. ENNAACT uses a neural network classifier based on sequence information to test anti-cancer activity of peptides [23]. We used standard settings and tested against the main dataset. Hemolytic activity was predicted applying HAPPENN with standard settings and the main dataset. HAPPENN utilizes deep neural networks to possibly separate hemolytic versus non-hemolytic peptides [24].

## Data Availability

Not applicable.
